# Alantolactone selectively ablates acute myeloid leukemia stem and progenitor cells

**DOI:** 10.1186/s13045-016-0327-5

**Published:** 2016-09-22

**Authors:** Yahui Ding, Huier Gao, Yu Zhang, Ye Li, Neil Vasdev, Yingdai Gao, Yue Chen, Quan Zhang

**Affiliations:** 1 State Key Laboratory of Medicinal Chemical Biology, College of Pharmacy and Tianjin Key Laboratory of Molecular Drug Research, Nankai University, Haihe Education Park, 38 Tongyan Road, Tianjin, 300353 People’s Republic of China; 2 State Key Laboratory of Experimental Hematology, Institute of Hematology and Hospital of Blood Diseases, Chinese Academy of Medical Sciences and Peking Union Medical College, Tianjin, 300020 People’s Republic of China; 3 Division of Nuclear Medicine and Molecular Imaging, Gordon Center for Medical Imaging, Massachusetts General Hospital, Boston, 02114 MA USA; 4 Department of Radiology, Harvard Medical School, Boston, MA USA

**Keywords:** Alantolactone, Acute myeloid leukemia stem cells, KG1a, Apoptosis

## Abstract

**Background:**

The poor outcomes for patients diagnosed with acute myeloid leukemia (AML) are largely attributed to leukemia stem cells (LSCs) which are difficult to eliminate with conventional therapy and responsible for relapse. Thus, new therapeutic strategies which could selectively target LSCs in clinical leukemia treatment and avoid drug resistance are urgently needed. However, only a few small molecules have been reported to show anti-LSCs activity.

**Methods:**

The aim of the present study was to identify alantolactone as novel agent that can ablate acute myeloid leukemia stem and progenitor cells from AML patient specimens and evaluate the anticancer activity of alantolactone in vitro and in vivo.

**Results:**

The present study is the first to demonstrate that alantolactone, a prominent eudesmane-type sesquiterpene lactone, could specifically ablate LSCs from AML patient specimens. Furthermore, in comparison to the conventional chemotherapy drug, cytosine arabinoside (Ara-C), alantolactone showed superior effects of leukemia cytotoxicity while sparing normal hematopoietic cells. Alantolactone induced apoptosis with a dose-dependent manner by suppression of NF-kB and its downstream target proteins. DMA-alantolactone, a water-soluble prodrug of alantolactone, could suppress tumor growth in vivo.

**Conclusions:**

Based on these results, we propose that alantolactone may represent a novel LSCs-targeted therapy and eudesmane-type sesquiterpene lactones offer a new scaffold for drug discovery towards anti-LSCs agents.

**Electronic supplementary material:**

The online version of this article (doi:10.1186/s13045-016-0327-5) contains supplementary material, which is available to authorized users.

## Background

Patients diagnosed with acute myeloid leukemia (AML) have poor outcomes, with <50 % overall survival rates for younger patients and a <1 year for older patients [1, 2]. Such patients are typically treated with chemotherapy and/or hematopoietic stem cell (HSC) transplantation [3]. Effective strategies for AML remission and cure are greatly needed for the treatments. Leukemia stem cells (LSCs) can give rise to either daughter or differentiated cells and are attributed for maintaining and perpetuating AML specifically leukemia initiation, resistance to chemotherapy, prognosis relapse, and deterioration [2]. Similar to HSCs, LSCs also have the features of self-renewal, multi-potent differentiation, and relative quiescence [4–6]. Many clinical chemotherapy drugs which target cell cycle have little potency to eliminate the LSCs because of its quiescent status. Moreover, the P-glycoprotein that could pump chemotherapy drugs out of cancer cells is overexpressed on the surface of LSCs [7], which leads to drug resistance. LSCs in AML patients are associated with the low remission rate, short disease-free and overall survival rate [8]. Abnormal regulation of some signal pathways, such as p53, NF-kB, ROS, Wnt/β-catenin, or Notch pathway, is considered to be critical to the pathogenesis of LSCs. The recovery of these signal pathways is used for elimination of LSCs [9–13]. Previous studies have revealed that the subpopulation with the surface antigens CD34^+^CD38^–^ can be regarded as LSCs [14–16]. Targeting and eliminating LSCs has been proposed as a promising strategy for treatment of AML to decrease the rate of relapse or drug resistance. However, small molecules that can selectively target LSCs are rare and still at early stages of development, for example, parthenolide (PTL) [17], niclosamide [18], dimethyl-aminoparthenolide (DMAPT) [19], fenretinide [20], 4-benzyl-2-methyl-1,2,4-thiadiazolidine-3,5-dione (TDZD-8) [21], AR-42 [22], 4-hydroxynonenal (HNE) [23], and micheliolide (MCL) [24].

Alantolactone, a naturally occurring eudesmane-type sesquiterpene lactone (SL), showed multi-function in several human cancer cell lines in vitro, such as inhibitory effect on cancer cell survival and regulating NF-kB signal pathway [25–27]. The goal of the present work is to evaluate the effects and mechanisms of alantolactone on LSCs. The result indicated that alantolactone exhibited high cytotoxicity both in primary AML cells and multi-drug resistant cells. Alantolactone inhibited cell proliferation and induced apoptosis in a dose-dependent manner in vitro. Interestingly, alantolactone significantly induced apoptosis of CD34^+^CD38^–^ cells in primary AML specimens isolated from blood samples of AML patients and exhibited little cytotoxicity against normal hematopoietic cells. Moreover, alantolactone inhibited AML colony formation in vitro. In a NOD/SCID mice xenograft assay, a water-soluble prodrug DMA-alantolactone, was shown to suppress tumor growth in vivo, and no apparent toxicity was observed. These studies demonstrated that alantolactone could selectively ablate LSCs with little effect on normal hematopoietic cells. To our knowledge, this is the first report of a eudesmane-type sesquiterpene lactone with anti-LSCs activity.

## Methods

### Materials

Cell culture medium reagents, MTT, and DMSO were purchased from Sigma Chemical Company (St. Louis, MA, USA). Rabbit polyclonal anti-human p65, XIAP, FLIP, Bax, Bcl-2, PARP, caspase-3, caspase-9, and β-actin antibodies were purchased from Cell Signaling Technology (Beverly, MA, USA). Fetal bovine serum (FBS) was purchased from Gibco (NY, USA). Cell lysis buffer was purchased from Beyotime Institute of Biotechnology (Beijing, China). AnnexinV-FITC and PI apoptosis detection kit was purchased from BD (BD, USA). Human CD34-APC, CD38-PE.cy7, CD33-APC, CD19-PE, CD235a-FITC, and CD3-APC were purchased from BD (BD, USA). ECL-Plus Kit was purchased from Thermo Scientific (Rockford, USA).

### The isolation of alantolactone and synthesis of DMA-alantolactone

The air-dried roots of *Inula helenium* L. (15.0 kg) were percolated with EtOH (3 × 25 L) at room temperature. The combined extracts were concentrated under reduced pressure. The resulting residue was partitioned into H_2_O and extracted with EtOAc. The EtOAc fraction was subjected to silica gel column chromatography (petroleum ether–EtOAc from 98:2 to 1:1, *v*/*v*) to give crude alantolactone. The crude residue was subjected to repeated CC over silica gel (petroleum ether–EtOAc, 10:1, *v*/*v*) and repeated column chromatography over Sephadex LH-20 (MeOH) to afford alantolactone (52 mg).

A mixture of Me_2_NH·HCl(263 mg, 3.2 mmol), K_2_CO_3_ (892 mg, 6.5 mmol), and CH_2_Cl_2_ (12 mL) was stirred at room temperature until Me_2_NH·HCl was dissolved. The solid in the mixture was filtered off, and the resulting solution was treated with alantolactone (50 mg, 0.22 mmol). The reaction mixture was stirred at room temperature for 3 h. The reaction mixture was concentrated under reduced pressure, and the residue was dissolved in CH_2_Cl_2_, and then the solution was washed with water. The organic layer was dried over anhydrous Na_2_SO_4_, concentrated under reduced pressure to give white solid (50.4 mg, 0.18 mmol). The solid, fumaric acid (21 mg, 0.18 mmol), and MeOH was stirred for 2 h. The mixture was concentrated to afford white product (71.4 mg). The yield of the two steps is 81.4 %. ^1^H NMR (400 MHz, D_2_O) δ 6.59 (s, 2H), 5.05 (d, *J* = 2.5 Hz, 1H), 4.94 (s, 1H), 3.55 (td, *J* = 8.5, 5.8 Hz, 1H), 3.45–3.32 (m, 2H), 3.27 (dd, *J* = 9.8, 6.8 Hz, 1H), 2.89 (s, 6H), 2.46–2.35 (m, 1H), 2.03 (dd, *J* = 15.2, 3.1 Hz, 1H), 1.74 (dd, *J* = 26.7, 13.3 Hz, 1H), 1.58–1.24 (m, 6H), 1.08 (s, 3H), 1.01 (d, *J* = 7.6 Hz, 3H); ^13^C NMR (100 MHz, D_2_O) *δ* 178.8, 171.0, 153.8, 134. 6, 112.5, 79.9, 54.3, 44.5, 42.4, 41.55, 41.3, 38.2, 37.4, 32.5, 32.3, 28.1, 22.0, 16.2; HRMS (ESI) calcd for C_17_H_27_O_2_ [M + H]^+^ 278.212, found 278.2120 (Additional file 1).

### Cell culture

Human leukemia cell lines THP-1, KG1a, HL60, K562, HL60/ADR, and K562/A02 were cultured in 1640 medium containing with 10 % fetal bovine serum at 37 °C, 5 % CO_2_ incubator. Mononuclear cells isolated from the primary human AML samples using Ficoll-Paque density gradient separation were cultured in serum-free IMDM medium for 1 h, and then the cells were cultured with different concentrations of alantolactone.

### Cytotoxicity assay

MTT assay was usually used to measure cell viability and cytotoxicity of anti-cancer drugs. Briefly, leukemia cells were seeded in 96-well plates (1 × 10^4^ cells/well). Then, cells were treated with various concentrations of alantolactone and control group was treated by DMSO. After 72-h treatment of alantolactone, 20 μL MTT solution (5 mg/mL) was added to each well and then incubated in 37 °C, 5 % CO_2_ incubator for additional 4 h. After being centrifuged by 1500 rpm for 15 min, all supernatant were removed and 100 μL DMSO was added to each well to dissolve the formazan crystal. Absorbance was measured at 570 nm using a micro-plate reader (synergy H4, BioTek, USA). Then the IC_50_ value was analyzed by GraphPad Prism 5 project.

### Apoptosis assay

Apoptosis was assayed by flow cytometry, and apoptosis status of KG1a cells was stained with APC-Annexin V and 7-aminoactinomycin (7-AAD) with Apoptosis Assay kit (BD, USA) according to the manufacturer’s protocol. Briefly, 1 × 10^5^ KG1a cells or 1 × 10^6^ primary AML mononuclear cells were seeded in six-well plate. After 1-h incubation, cells were treated by different concentration of alantolactone for 24 or 18 h, and then cells were harvested and washed by cold PBS for three times. Cells were re-suspended with 1× binding buffer, and 5 μL APC-Annexin V and 5 μL 7-AAD were added to stain cells. After incubation 15 min in the dark, cells were analyzed by flow cytometry.

### Differentiation assay

AML mononuclear cells were obtained from AML samples by density gradient centrifugation. CD34^+^ AML cells were enriched by magnetic-activated cell sorting CD34 progenitor kit (Miltenyi Biotech, Auburn, CA, USA). After enriched, CD34^+^ cells were cultured in IMDM supplemented with 10 % fetal calf serum. Cytokines were added at the concentration of 100 ng/mL for rhSCF, 100 ng/mL for rhFlt3, and 100 ng/mL for rhTPO. Then cells were treated with various concentrations of alantolactone. After 3 days, cells were collected and stained with CD19-PE, CD33-APC, CD3-APC, and CD235a-FITC for 30 min, respectively. The cells were re-suspended and analyzed by flow cytometry.

### LSCs apoptosis assay in AML samples

The effect of alantolactone on LSC cells was analyzed by flow cytometry. The primary AML mononuclear cells were obtained from AML patients. The mononuclear cells which was isolated from primary AML patients were seeded in 24-well plates (1 × 10^6^ cells/well), and medium volume was 1 mL. Then, cells were treated with various concentrations of alantolactone. After 18-h treatment, the cells were re-suspended with PBS and stained with CD34-APC and CD38-PE.cy7 for 30 min, and then the cells were re-suspended with 1× binding buffer which contained 5 μL Annexin-V-FITC and 5 μL PI. Samples were analyzed by flow cytometry in 1 h.

### Clonogenic assay

Mononuclear cells were cultured in serum-free IMDM medium for 18 h in the presence or absence of alantolactone or Ara-C. Cells were plated at 200,000 cells/mL in MethoCult H4434 (stem cell). Cells were cultured for 14 days, and the number of colonies formed was counted under microscope.

### Acute toxicity assay in Kunming mice

Five 5-week-old Kunming mice (Chinese Academy of Sciences, Shanghai, China) were dosed orally with DMA-alantolactone at a single dose of 500 mg/kg. After administration, the mice were observed for their behavior and appetite. The body weight of the mice was weighed every day.

### Nude mice tumor growth inhibition assay

KG1a cells were collected and washed with PBS once. Then KG1a cells (1 × 10^7^ cells/0.1 mL PBS) were injected into 5-week-old female BALB/c nude mice (Chinese Academy of Sciences, Shanghai, China). After 20 days, tumor volume reached to 500 mm^3^, and tumor was cut to the same volume (0.5 mm^3^). And then small tumor pieces were implanted into mice. Tumor volumes and weights were measured every 3 days with an automatic vernier caliper. When tumor volume reached to 100 mm^3^, DMA-alantolactone was administrated by oral every 3 days with 100 mg/kg. Mice were sacrificed after 30 days. Tumor growth curves and growth inhibition rates were calculated. Five mice were used for each experimental group and control group. Animal assay was performed according to national and international guidelines, and mice were cultured in the Animal Ethics Committee of the Institute of Hematology & Hospital of Blood Diseases, Chinese Academy of Medical Sciences & Peking Union Medical College.

### Western blot assay

KG1a, HL60, and primary CD34^+^ AML cells were harvested after treatment by different concentrations of alantolactone for 12 h, re-suspended in cell lysis buffer RIPA (Beyotime, China) with 1 % phenylmethanesulfonyl fluoride (PMSF) and then incubated on ice for 30 min. Cell supernatants were mixed with loading buffer and boiled for 10 min at 100 °C, and protein samples were separated by SDS-PAGE in a 12 % gel. After electrophoresis, proteins were transferred into nitrocellulose (NC) membrane and blocked with 5 % non-fat milk powder in PBST buffer (PBS with 0.05 % Tween-20) for 1 h at room temperature. The membrane was incubated with diluted primary antibody at 4 °C overnight. The membrane was washed with PBST for three times and then incubated with HRP-conjugated secondary antibody for 2 h at room temperature. Finally, proteins were assayed by an ECL-Plus Kit.

### Statistical analysis

Results were repeated more than three independent experiments and each experiment was done in triplicate. Student’s *t* test was performed with GraphPad Prism5 software to analyze the significance level, and the *P* value of less than 0.05 was considered to be statistically significant. Error bars denoted the standard deviation (SD), unless stated otherwise.

## Results

### Alantolactone inhibited the proliferation of different leukemia cells in vitro

**Fig. 1:**
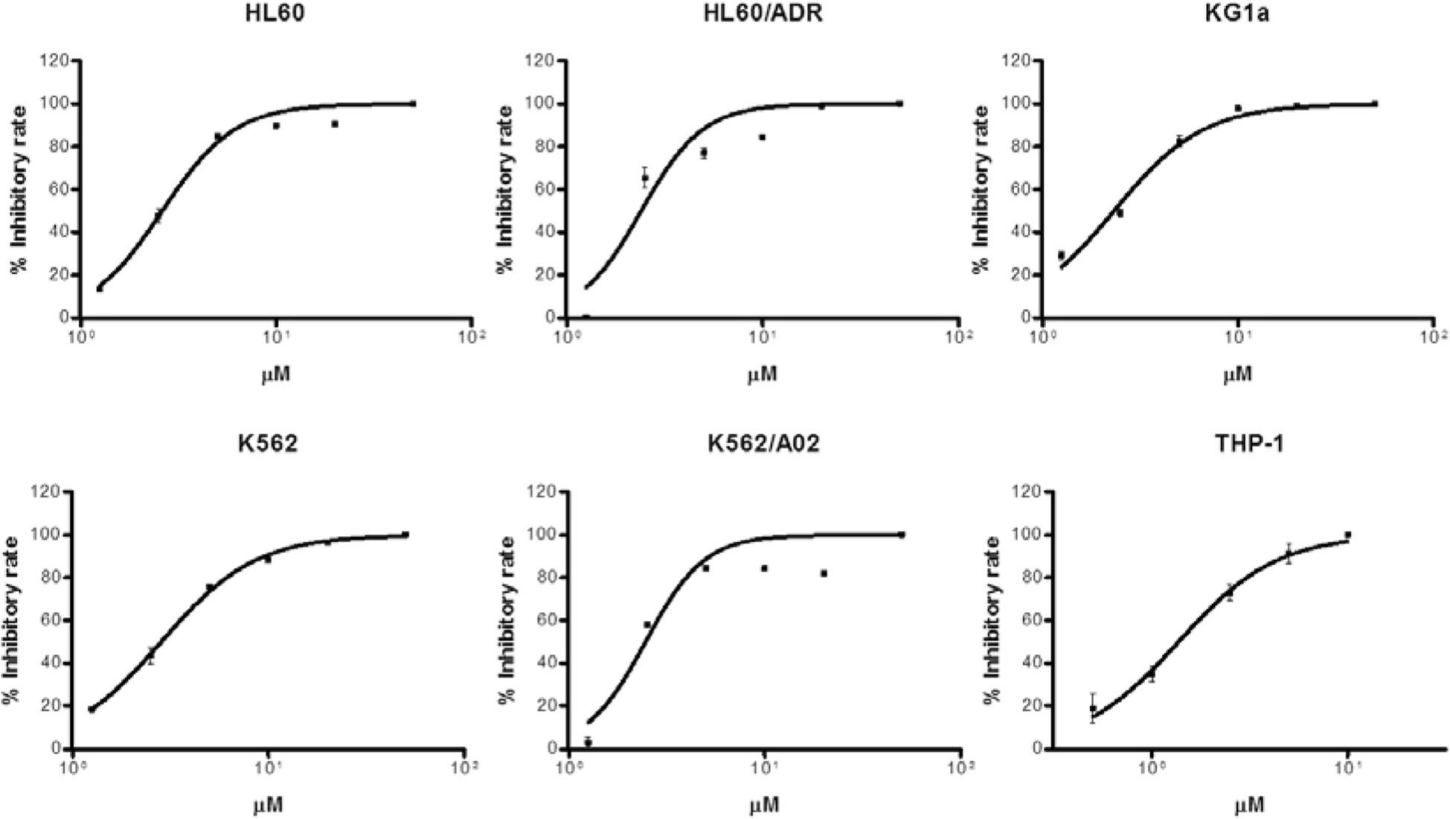
Cytotoxicity of alantolactone against different leukemia cell lines. Cytotoxicity was assayed by MTT. HL60, HL60/ADR, KG1a, K562, K562/A02, and THP-1 cells were treated with a range dose of alantolactone (1.25, 2.5, 5, 10, 20, and 50 μM) for 72 h, respectively. Numerical analysis data represented mean ± SD based on three independent experiments

Initial studies were performed to examine the cytotoxicity of alantolactone in different leukemia cell lines in vitro, including human leukemia cell lines HL60, K562, their multidrug-resistant counterparts HL60/ADR, K562/A02, respectively, and a human AML cell line, THP-1 (Fig. 1, Table 1). All leukemia cells were cultured with alantolactone for 72 h. Alantolactone showed significant inhibitory effect on the HL60 and K562 cells with IC_50_ values of 3.26 and 2.75 μM, respectively. Furthermore, the activities against drug-resistant cell lines HL60/ADR (IC_50_ = 3.28 μM) and K562/A02 = 2.73 μM) were comparable to their counterparts the sensitive cell lines HL60 and K562, while ADR, a clinically used drug, exhibited 205- to 59-fold IC_50_ values for the drug-resistant cell lines HL60/ADR and K562/A02 comparing to those of the sensitive cell lines HL60 and K562, respectively. Alantolactone was active against human AML cell line THP-1 with IC_50_ value of 2.17 μM. Alantolactone demonstrated anti-cancer activity with a dose-dependent manner in different leukemia cell lines.

**Table 1:** Inhibitory effect of alantolactone on different leukemia cell lines

Cell lines	IC_50_ (μM)^a^	
	Alantolactone	ADR
K562	2.75 ± 0.64	0.15 ± 0.08
K562/A02	2.73 ± 0.83	8.94 ± 3.79
HL60	3.26 ± 0.88	0.027 ± 0.022
HL60/ADR	3.28 ± 0.80	5.54 ± 1.21
THP-1	2.17 ± 0.72	0.06 ± 0.038
KG1a	2.75 ± 0.65	0.62 ± 0.15
Normal cell	26.37 ± 4.04	N/A

*ADR* adriamycin, used as a positive control

^a^All values are the mean of three independent experiments

Previous studies indicated that KG1a cell line is a type of AML progenitor cell line which showed high multidrug resistance and self-renewal potential. Furthermore, KG1a cells had a large portion of cells bearing a CD34^+^CD38^–^ immunophenotype which was considered to be characteristic of LSCs. Therefore, KG1a was considered to be a LSCs-like cell line and an ideal cell line for preliminary screening of potential anti-AML stem cell agents [28–30]. We further examined the potency of alantolactone against KG1a. It is noteworthy that alantolactone showed high growth inhibition to KG1a (IC_50_ = 2.75 μM), which is comparable to that against HL60 and HL60/ADR.

In view of the high activity against AML cells, alantolactone was further studied to evaluate its selectivity against AML cells over normal cells. Normal hematopoietic cells were collected from healthy donors. The result indicated that alantolactone did not show significant effect on the viability of normal cells with IC_50_ value of 26.37 μM (Table 1). The selectivity index for KG1a and normal cells was 9.6, which suggested that alantolactone could selectively eliminate AML cells (IC_50_ = 2.75 μM for KG1a) with relatively low toxicity against normal hematopoietic cells (IC_50_ = 26.37 μM).

### Alantolactone induced apoptosis and differentiation of stem-like cells

**Fig. 2:**
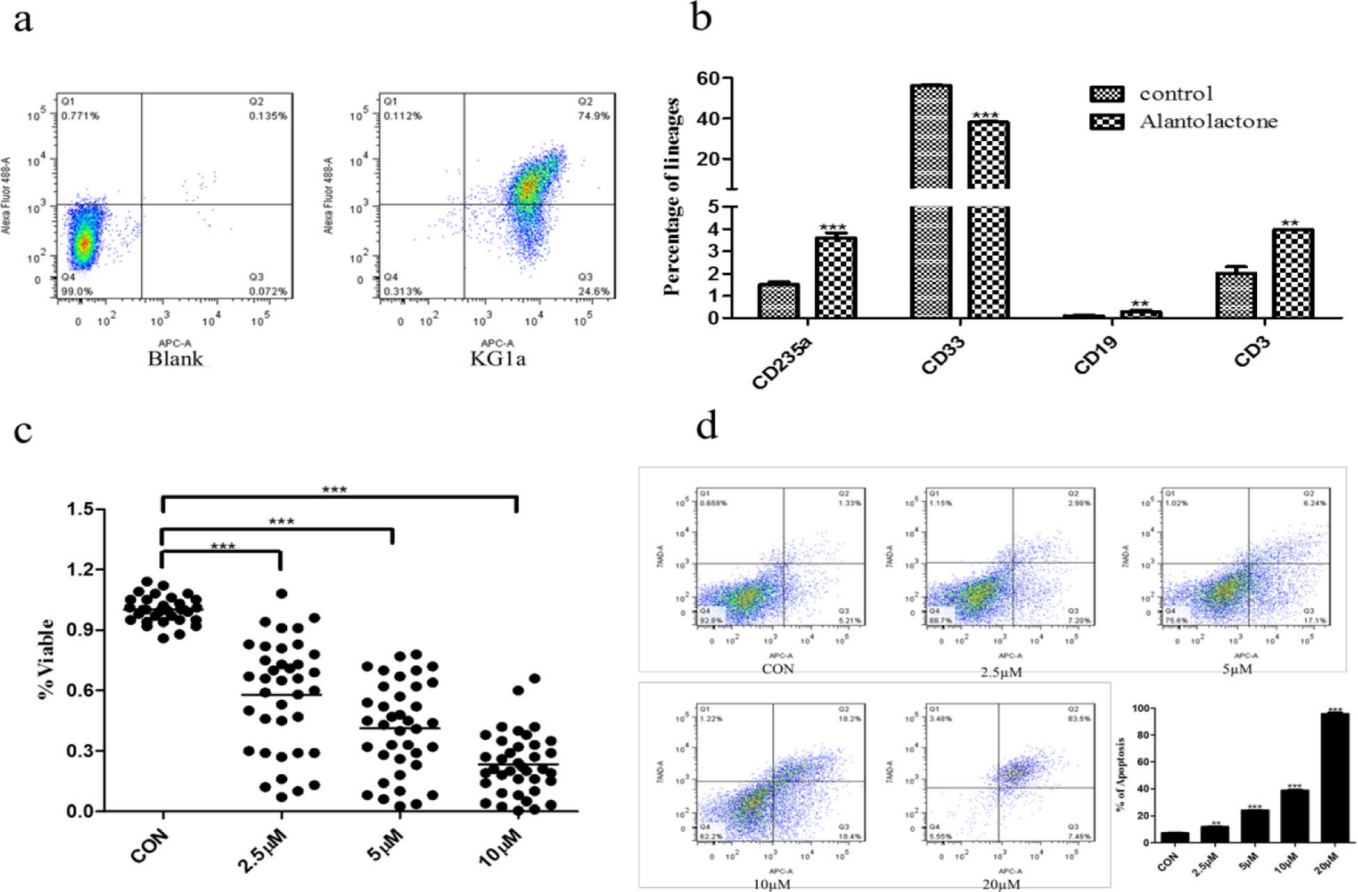
Alantolactone induced the apoptosis and differentiation of leukemia stem-like cell line KG1a and primary AML cells. **a** The expression of CD34^+^CD38^–^ on the surface of KG1a cells by flow cytometry. The *χ* axis represented CD34-APC, and the *Y* axis represented CD38-AF488. **b** Alantolactone induced the differentiation of leukemia stem cells after 3 days treatment. **c** Alantolactone induced apoptosis of primary AML cells with a dose-dependent manner after the treatment of alantolactone for 18 h. Each point represents an AML specimen. **d** Alantolactone induced apoptosis with a dose-dependent manner in KG1a cells with the treatment of alantolactone for 24 h. Analysis results represented mean ± SD based on three independent experiments, **P < 0.01, ***P < 0.0001

We examined the population of LSCs in the KG1a cell line, which was reported to contain 54 % CD34^+^CD38^–^ cells [28]. From our result of flow cytometry, the percentage of CD34^+^ cells in KG1a cell line was 99.3 %, and the percentage of CD34^+^CD38^–^ was 37.4 % (Fig. 2a), confirming that KG1a represented a AML stem-like cell line. In differentiation assay, alantolactone resulted in a significant increment in the frequency of lymphocyte cell (CD19), T cell (CD3), and erythroid cell (CD235a) and a significant decrease in the frequency of myeloid cell (CD33) (Fig. 2b). Alantolactone exerted a dose-dependent cytotoxic effect on total primary AML cells from AML specimens (Fig. 2c). Furthermore, apoptosis was determined by APC Annexin V and 7-AAD staining and was assessed by flow cytometry. Alantolactone induced apoptosis in KG1a cells with a dose-dependent manner. After culturing with alantolactone for 24 h, the apoptotic percentage of KG1a was 10.9 ± 0.35 % (2.5 μM), 20.8 ± 3.12 % (5 μM), and 36.0 ± 1.56 % (10 μM) comparing with negative control 8.27 ± 0.92 % (DMSO) in KG1a cells (Fig. 2d).

### Alantolactone specifically induced apoptosis of primary AML stem and progenitor cells while sparing normal hematopoietic stem and progenitor cells

**Fig. 3:**
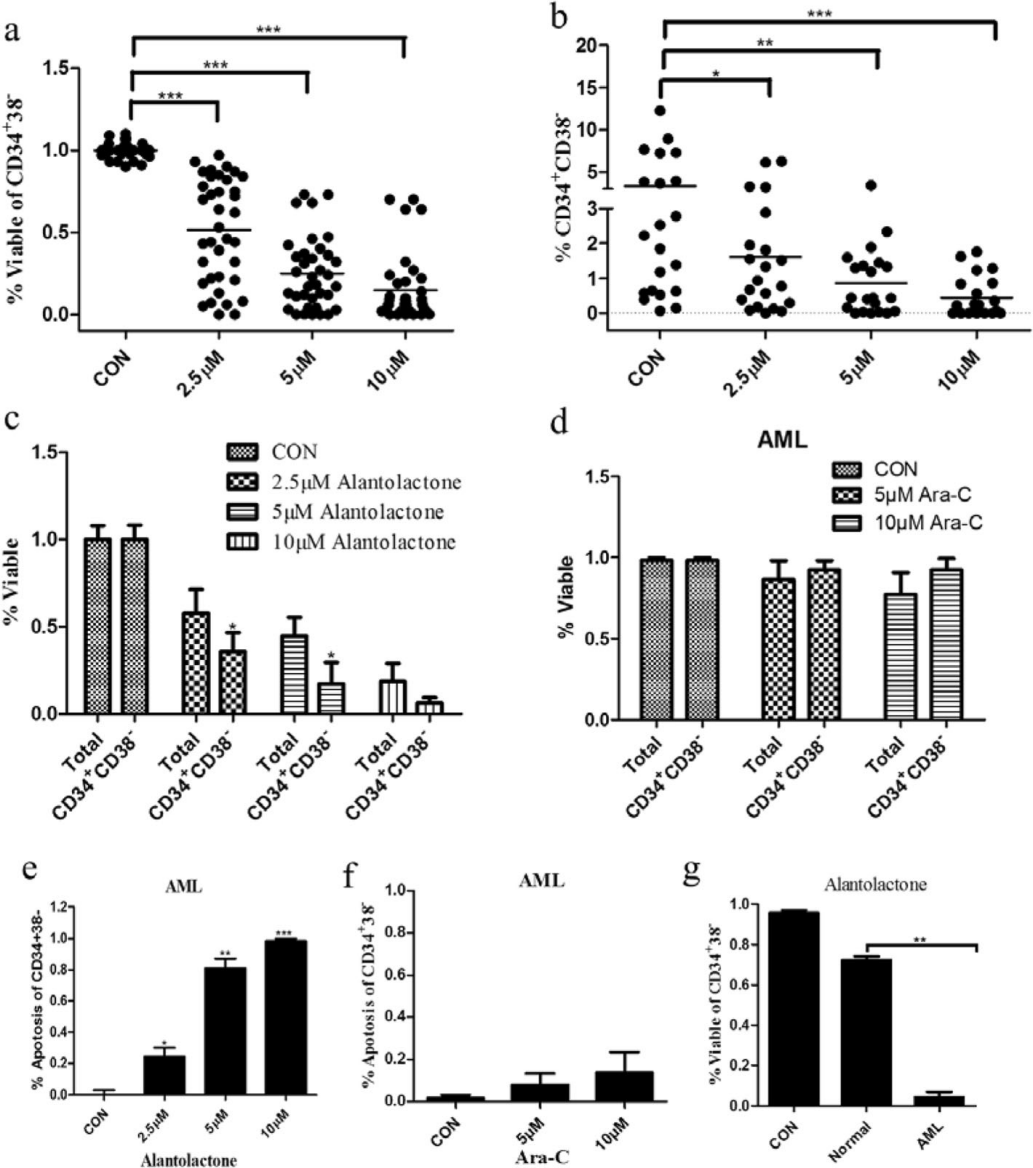
Alantolactone selectively induced apoptosis of LSCs in primary AML cells. **a** Alantolactone induced the apoptosis of CD34^+^CD38^–^ cells with a dose-dependent manner in primary AML cells from AML specimens after treatment of alantolactone for 18 h. Each point represents an AML specimen. **b** Alantolactone reduced the percentage of CD34^+^CD38^–^ cells with a dose-dependent manner in primary AML cells after the treatment of alantolactone for 18 h. Each point represents an AML specimen. **c** Alantolactone selectively induced apoptosis of CD34^+^CD38^–^ cells comparing with total cells in primary AML cells after the treatment of alantolactone for 18 h by flow cytometry. **d** Ara-C showed negligible cytotoxicity to CD34^+^CD38^–^ and total cells in primary AML cells at the concentrations of 5 and 10 μM. **e** Alantolactone induced apoptosis of CD34^+^CD38^–^ cells in primary AML cells by flow cytometry at the concentrations of 2.5, 5, and 10 μM. **f** Ara-C showed almost no effect on inducing apoptosis of CD34^+^CD38^–^ cells in primary AML cells at the concentrations of 5 and 10 μM. **g** Alantolactone treatment demonstrated significantly less effect on inducing apoptosis of CD34^+^CD38^–^ cells in normal hematopoietic cells comparing to that in primary AML cells at a concentration of 10 μM. *P < 0.05, **P < 0.01, ***P < 0.0001

In previous study, alantolactone showed potent anti-cancer activity [25–27]; however, there was no study on the effect of alantolactone on LSCs. To investigate the effects of alantolactone on selectively targeting LSCs, we initially studied the effects of alantolactone on primary leukemia specimens during suspension culture. The result is described in Table 2. The viability of the cells, bearing a CD34^+^CD38^–^ immunophenotype which were considered to be characteristic of LSCs, was determined after treatment with 2.5, 5, and 10 μM alantolactone for 18 h. At a concentration of 2.5 μM, three of the specimens, AML9, AML12, and AML13, showed excellent response with very low viability (0, 6.6, and 6.2 % viable CD34^+^CD38^–^ cells, respectively). The viability of most of specimens was greatly reduced by increasing concentration of alantolactone to 10 μM, with the exception of AML2, AML6, AML14, AML15, and AML18 with >25 % viable CD34^+^CD38^–^ cells. Alantolactone induced apoptosis of primary AML CD34^+^CD38^–^ cells with a dose-dependent manner (Fig. 3a, e). Moreover, the percentage of CD34^+^CD38^–^ cells in total viable cells was also decreased (Fig. 3b).

**Table 2:** Viability of primary AML cells treated with alantolactone

AML specimens	Viability of CD34^+^CD38^−^ (%)^a^		
	2.5 μM	5 μM	10 μM
AML1	67.9	10.0	2.1
AML2	74.5	62.1	43.7
AML3	75.9	19.6	1.5
AML4	17.9	10.4	8.9
AML5	58.8	46.3	24.7
AML6	85.4	70.6	66.9
AML7	40.3	9.6	4.6
AML8	86.1	29.8	3.2
AML9	0.0	0.0	0.0
AML10	19.6	15.0	7.2
AML11	53.3	6.4	5.0
AML12	6.6	3.2	2.1
AML13	6.2	2.0	0.0
AML14	35.4	28.4	25.5
AML15	91.7	31.6	26.6
AML16	76.3	35.3	6.0
AML17	37.5	28.9	8.9
AML18	85.5	70.5	67.0

All values were carried out as triplicate

^a^The percentage of viability was normalized to untreated control

For analysis of the effect of alantolactone selectively targeting LSCs, we analyzed and compared the viability of both the total and CD34^+^CD38^–^ cells. As shown in Fig. 3c, alantolactone showed high toxicity to both of the total and CD34^+^CD38^–^ cells. It is notable that CD34^+^CD38^–^ cells were more sensitive to alantolactone than the total primary cells.

To further evaluate the potency of alantolactone, we also studied the efficacy of the standard chemotherapy drug Ara-C on CD34^+^CD38^–^ cells from AML specimen for comparison. Ara-C demonstrated very little toxicity to total primary AML cells, especially to CD34^+^CD38^–^ cells with almost no efficacy (Fig. 3d, f). Both of normal HSCs and LSCs are with characteristic of bearing CD34^+^CD38^–^ immunophenotype. Therefore, we further evaluated the cytotoxicity of alantolactone against normal hematopoietic stem cells from the umbilical cord. As shown in Fig. 3g, normal CD34^+^CD38^–^ cells showed relatively less apoptotic response to alantolactone treatment comparing with the response to leukemia stem cells at 10 μM. Taken together, these results demonstrated that alantolactone had specific toxicity against LSCs while relatively less toxicity to normal HSCs.

### Alantolactone suppressed AML but not normal colony formation

**Fig. 4:**
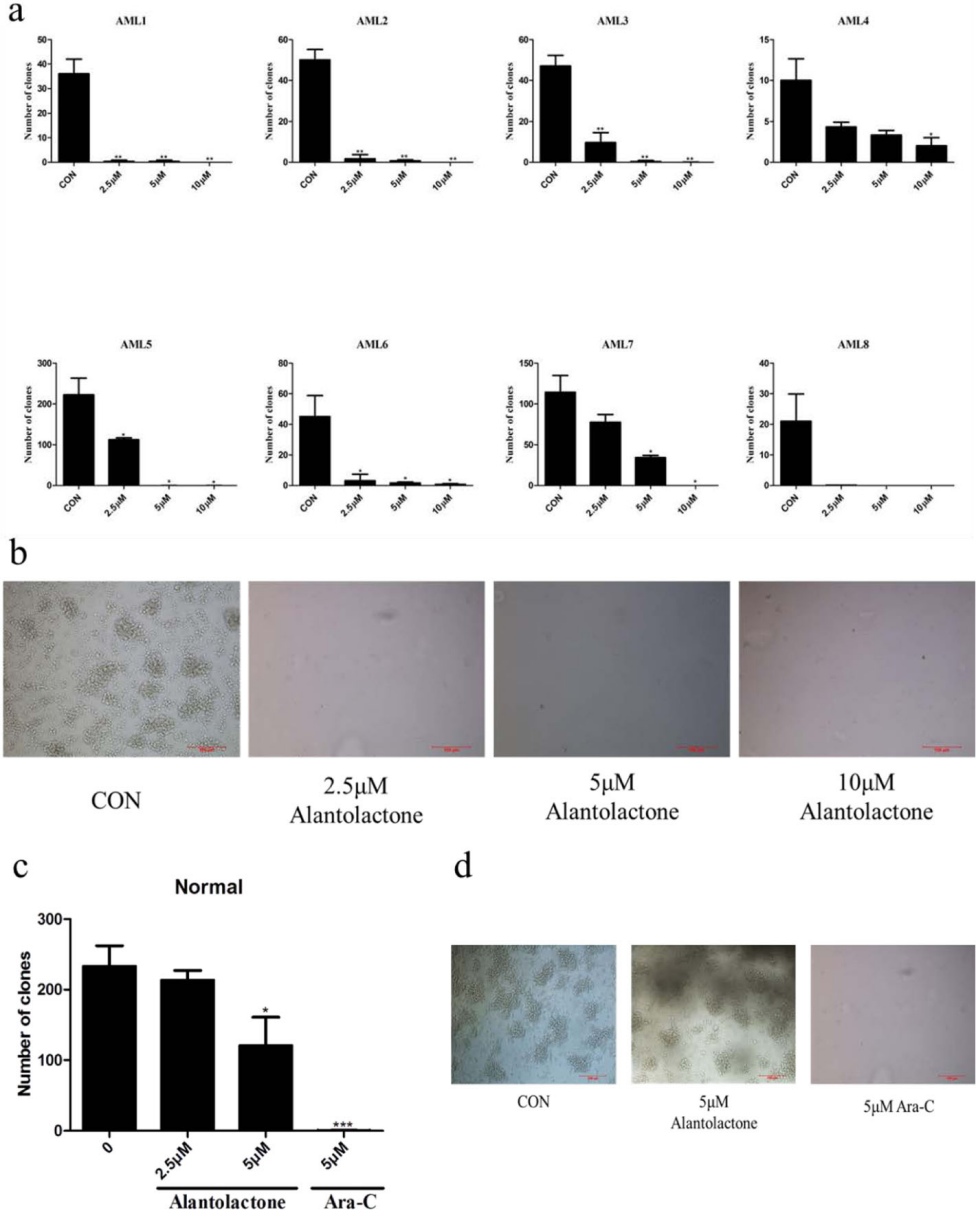
Alantolactone suppressed primary AML but not normal colony formation after 14 days in MethoCult H4434. **a** Alantolactone dramatically reduced the number of CFUs in primary AML cells from eight AML specimens with a dose-dependent manner. **b** The representative microscopy images of CFUs in primary AML cells were shown. **c** Alantolactone showed little effect on the CFUs in normal hematopoietic cells and Ara-C treatment led to notable reduction of colonies from normal donors. **d** The representative microscopy images of CFUs in normal hematopoietic cells were shown. *P < 0.05, **P < 0.01, ***P < 0.0001

Colony formation assay was conducted to determine the effect of alantolactone on the potential of primitive cells. After treatment with alantolactone, the number of colony-forming units (CFUs) of AML stem/progenitor cells significantly decreased comparing with the control group in a dose-dependent manner (Fig. 4a, b). Moreover, CFUs of normal hematopoietic cells from the umbilical cord showed a little effect. In contrast, normal colony formation was greatly reduced at a concentration of 5 μM Ara-C (Fig. 4c, d). Comparing with Ara-C, alantolactone presented significant selectivity on colony formation of primary AML cells. The result indicated that alantolactone treatment affected AML but not normal colony formation.

### Alantolactone effectively inhibited tumor growth in vivo

**Fig. 5:**
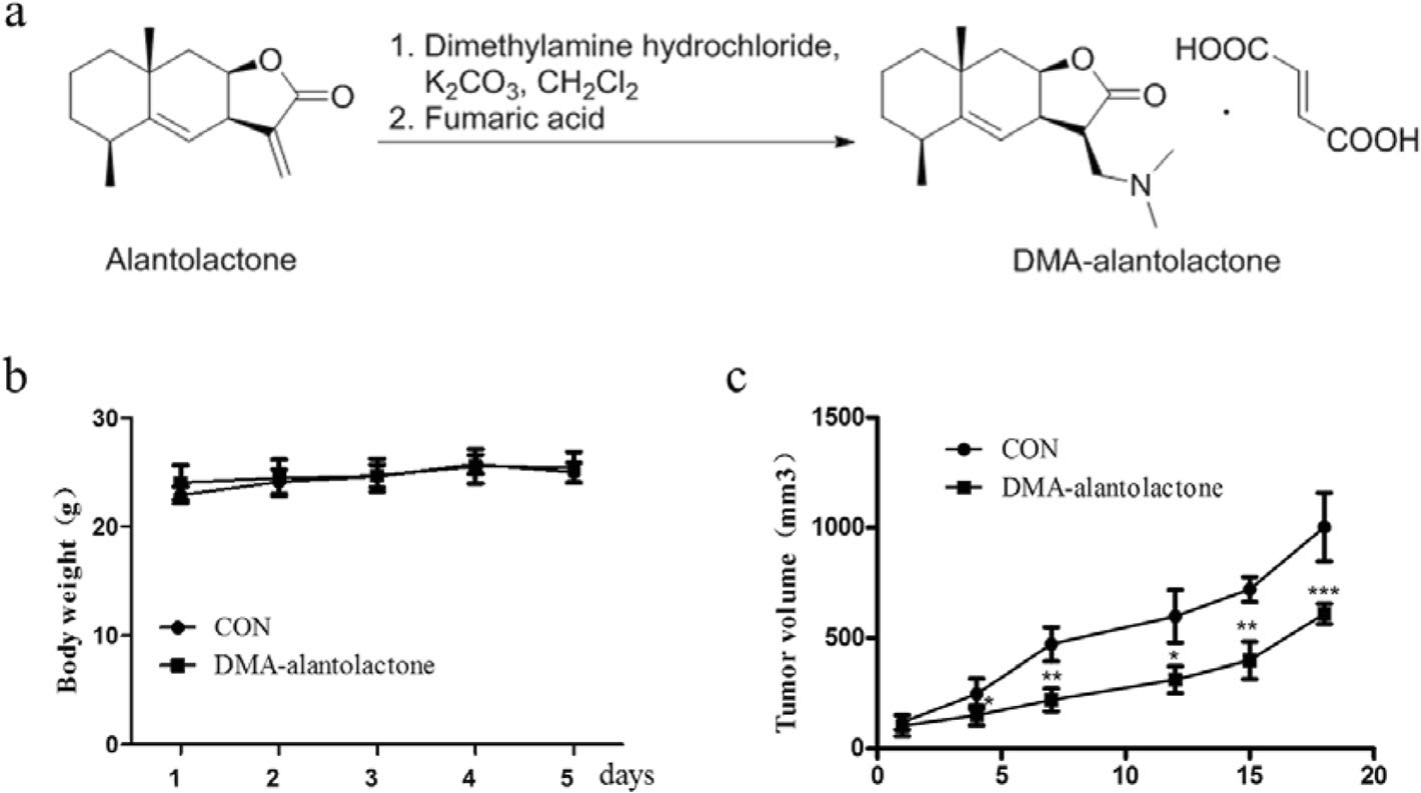
DMA-alantolactone inhibited tumor growth effectively in vivo. **a** Synthesis of the pro-drug of alantolactone, i.e., DMA-alantolactone. **b** The body weight of the mice after the oral administration of alantolactone at a dose of 500 mg/kg. **c** KG1a cells were injected into 5-week-old female BALB/c nude mice. After 20 days, tumor was cut to the same volume (0.5 mm^3^). The small tumor pieces were implanted into mice. When tumor volume reached to 100 mm^3^, DMA-alantolactone was administrated by oral every 3 days with 100 mg/kg. Mice were sacrificed after 30 days. *P < 0.05, **P < 0.01, ***P < 0.0001

Taking into account that alantolactone showed significant cytotoxicity against AML cells in vitro, we planned to evaluate alantolactone for its anti-AML activity in vivo. However, alantolactone showed low solubility in water. Therefore, alantolactone was converted to its prodrug, DMA-alantolactone (Fig. 5a). Firstly, we evaluated the acute toxicity of DMA-alantolactone in normal Kunming mice. As shown in Fig. 5b, the body weight of mice was not apparently changed after a single oral administration of 500 mg/kg. All mice remained alive, and no significant side effects were observed. When tumor volumes reached to 100 mm^3^, DMA-alantolactone was administrated and data presented that tumor sizes were significantly suppressed with comparing to control group (Fig. 5c). Moreover, weight of the mice did not change obviously. The result demonstrated that DMA-alantolactone could greatly inhibit tumor growth in vivo.

### Alantolactone induced apoptosis by the suppression of NF-kB and its downstream target proteins

**Fig. 6:**
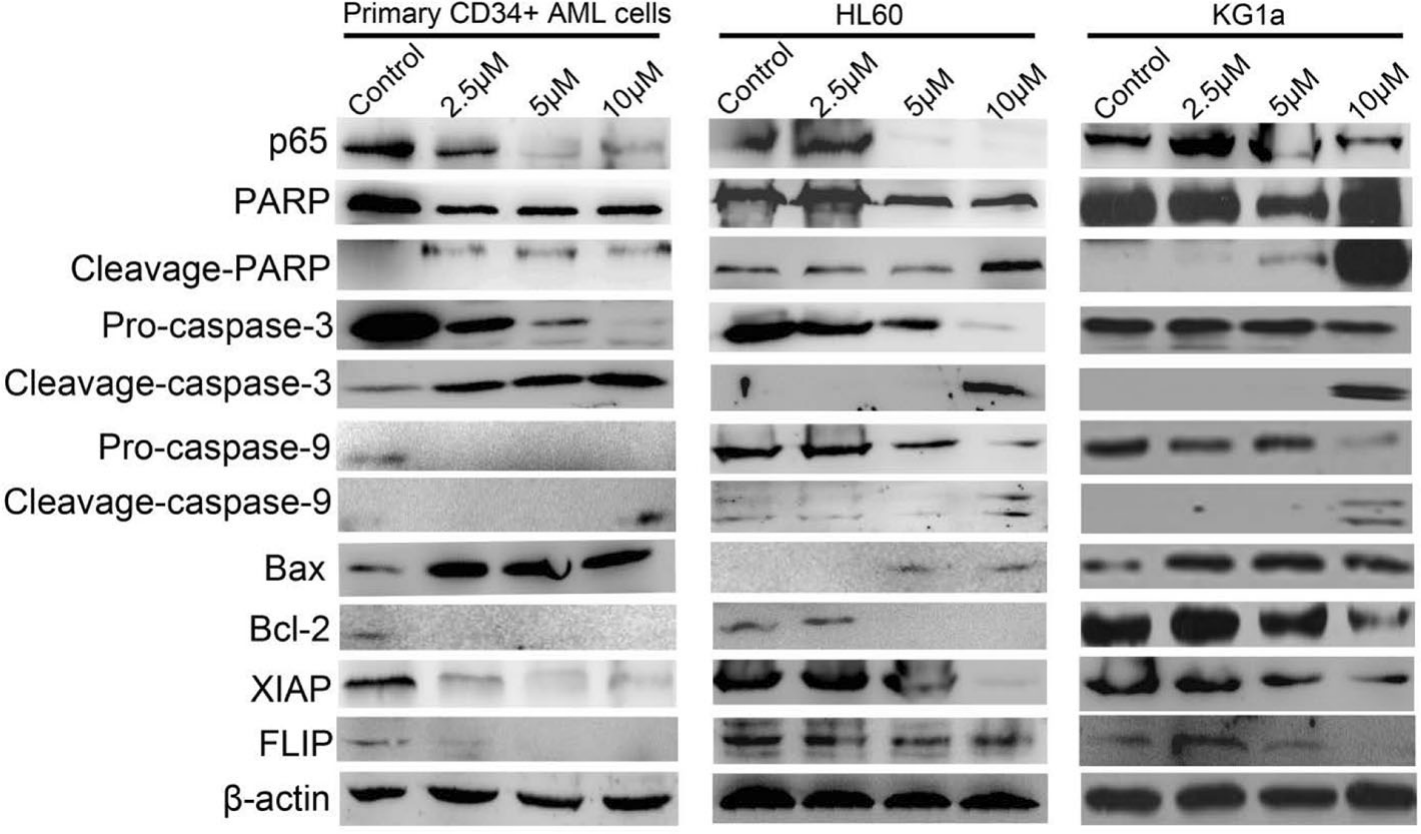
Alantolactone induced apoptosis by the suppression of NF-kB and its downstream target proteins. The expression of p65, XIAP, FLIP, Bcl-2, Bax, and the cleavage of PARP, caspase-3, caspase-9 was detected by western blot assay after the treatment of alantolactone for 12 h in primary CD34^+^ cells, HL60, and KG1a cells

For exploring the way of alantolactone inducing apoptosis, we assayed the status of apoptosis by Western blot analysis of Bcl-2, Bax, caspase-3, caspase-9, p65, XIAP, FLIP, and PARP in primary CD34^+^, HL60, and KG1a cells. Bcl-2 family played an important role in the regulation of apoptosis, and the expression level of Bcl-2 and Bax proteins were usually assayed for characteristic of apoptosis. After 24-h treatment of alantolactone, the expression of Bcl-2, the inhibitors of apoptosis, was significantly reduced and the expression of Bax, the pro-apoptotic protein, was increased comparing with control group (Fig. 6). Caspase-3, caspase-9, and PARP were major factors in cell death cascade. We examined the effects of alantolactone on the activation of caspase-3, caspase-9, and PARP. Cleavages of caspase-3, caspase-9, and PARP were increased with treatment of alantolactone. The expression of p65 which played important roles in NF-kB signal pathway decreased significantly. Meanwhile, the levels of XIAP and FLIP, the downstream target proteins of NF-kB, were reduced simultaneously after the treatment of alantolactone.

## Discussion

AML is one of the most common forms of malignant myeloid leukemia in adults. There are several strategies in clinical treatments for younger adults. However, it remains a challenge to increase the survival and cure rates for patients who are older than 70 years [31]. Drug resistance and recurrence are the main obstacles for treatment of AML. Finding new anti-AML agents or targeting AML therapies is presently an active area for clinical trials [2]. A small population of AML cells with self-renewal function is considered to be LSCs. It has been reported that LSCs which bear the CD34^+^CD38^–^ immunophenotype play the major role for initiation of leukemia, drug resistance, and recurrence of leukemia, and are related to minimal residual disease (MRD) [32, 33]. Selectively targeting LSCs is postulated to prevent tumor relapse and drug resistance without damage of the normal hematopoietic stem cells.

Alantolactone, a sesquiterpene lactone isolated from *I. helenium* L., has been studied as a potential anticancer agent in several different cancer lines [34–36]. Alantolactone could reduce P-glycoprotein expression in drug resistant cell line K562/adriamycin for drug resistance treatment [37]. It has been reported to obtain alantolactone by separating from Chinese herbs [38]. Herein, our research first reveals that alantolactone can selectively target LSCs.

Alantolactone exhibited comparable cytotoxicity to drug-resistant cell lines K562/A02, HL60/ADR with drug sensitive cell lines K562 and HL60 (Fig. 1, Table 1). It was reported that LSCs showed the character of multi-drug resistance. The cell line KG1a, which exhibited high P-glycoprotein-mediated drug efflux capacity and a high level of DNR resistance, showed a strong response to alantolactone treatment. The previous work [28] and the present study indicated that KG1a, bearing a high level of CD34^+^ and CD34^+^CD38^–^, was a stem-like cell line (Fig. 2). We hypothesized that such agents that suppress drug-resistant cells would lead to a positive response to cancer stem-like cells. Alantolactone exerted anti-AML activity by inducing apoptosis of AML cells. We assayed the apoptosis of primary AML cells from clinical specimens. Among 18 primary AML samples, the viability of CD34^+^CD38^–^ cells from most of specimens was greatly reduced with treatment of alantolactone at a concentration of 10 μM (Table 2). Furthermore, alantolactone was more toxic to CD34^+^CD38^–^ cells than total primary leukemia cells. In contrast, the traditional chemotherapy drug Ara-C exhibited little apoptosis when tested against total primary leukemia cells abf CD34^+^CD38^-^ cells at concentrations of 5 and 10 μM (Fig. 3). It is noteworthy that CD34^+^CD38^–^ cells of normal hematopoietic cells from the umbilical cord were almost unaffected with treatment of alantolactone at concentrations of 2.5 and 5 μM (Fig. 3). To further confirm the inhibitory effect of alantolactone on colony formation for primary AML cells, a colony formation assay was performed. Alantolactone significantly inhibited the number of CFUs in AML cells at a concentration of 2.5 μM, while the number of CFUs was little affected in normal hematopoietic cells at the same concentration (2.5 μM). In contrast, Ara-C strongly inhibited the number of CFUs in normal hematopoietic cells at the concentration of 5 μM (Fig. 4).

The above-mentioned experiments revealed that alantolactone exhibited significant anti-AML activity in vitro, which promted us to carry out in vivo experiments with the KG1a, an AML stem-like cell line. The prodrug of alantolactone, i.e., DMA-alantolactone, was evaluated in vivo (Fig. 5a). Acute toxicity assay indicated that DMA-alantolactone had little toxicity to normal mice with a single dose of 500 mg/kg (Fig. 5b). Meanwhile, DMA-alantolactone greatly inhibited the tumor size after the treatment comparing with control group. DMA-alantolactone could suppress tumor growth in vivo (Fig. 5c).

The expression of NF-kB in normal hematopoietic stem cells was low, while it was overexpressed significantly in leukemia stem cells [39]. Therefore, the expression and activity inhibition of NF-kB could be a potential therapeutic target for targeting leukemia stem cell. The expression of NF-kB and NF-kB-regulated proteins such as p65, Bcl-2, XIAP, and FLIP played important roles in cell apoptosis. It was reported that parthenolide and micheliolide, with chemically reactive alpha-beta unsaturated lactone moiety, were able to covalently bind to proteins via the Michael acceptor and eliminate leukemia stem cell by inhibiting the activity of NF-kB [17, 19, 39]. We speculated that compounds with alpha-beta unsaturated lactone moiety might share some common protein targets. Alantolactone also contained the moiety of alpha-beta unsaturated lactone, which prompted us to suppose that alantolactone would target AML stem cells by inhibiting the activity of NF-kB. The primary studies for mechanism of the anti-AML stem cell activity of alantolactone were performed. After the treatment of alantolactone, the expression of Bcl-2, p65, XIAP, and FLIP reduced and the expression of Bax increased obviously comparing with control group. Increasing cleavages of PARP, caspase-3, and caspase-9 were observed with the treatment of alantolactone (Fig. 6). These results indicated that alantolactone induced the apoptosis of AML stem cells mainly by the suppression of NF-kB and its downstream target proteins.

It is worth to note that the yield of alantolactone in *I. helenium* L. was extremely low, which would hamper the development of alantolactone as an anti-AML agent. We identified that isoalantolactone is the major constituent of *I. helenium* L. (0.6 %), and the structure of isoalantolactone is similar to alantolactone. Therefore, we proposed to convert isoalantolactone to alantolactone to circumvent the hurdle, and the study is ongoing in our lab.

## Conclusions

In summary, alantolactone, a prominent naturally occurring eudesmane-type sesquiterpene lactone, was firstly demonstrated to be a potential agent that can selectively target LSCs with negligible toxicity towards normal cells. Alantolactone induced apoptosis of AML stem cells by the suppression of NF-kB and its downstream target proteins. DMA-alantolactone, a water-soluble prodrug of alantolactone, was shown to inhibit tumor growth in vivo. Based on these results, we propose that alantolactone might be used as a potential LSC-targeted agent, and eudesmane-type sesquiterpene lactones represent a new scaffold for drug discovery of novel anti-LSCs agents.

## Additional file

Additional file 1: Copies of NMR, MS, HPLC spectra of alantolactone and copies of NMR spectra of DMA-alantolactone. (DOCX 533 kb)

## Abbreviations

7-AAD: 7-Aminoactinomycin
ADR: Adriamycin
Ara-C: Cytosine arabinoside
CFUs: Colony-forming units
DMAPT: Dimethyl-aminoparthenolide
HNE: 4-Hydroxynonenal
HSCs: Hematopoietic stem cells
LSCs: Leukemia stem cells
MCL: Micheliolide
MRD: Minimal residual disease
MTT: Methyl thiazolyl tetrazolium
PTL: Parthenolide
SL: Sesquiterpene lactone
TDZD-8: 4-Benzyl-2-methyl-1,2,4-thiadiazolidine-3,5-dione
