# Correction to: Alantolactone selectively ablates acute myeloid leukemia stem and progenitor cells

**DOI:** 10.1186/s13045-021-01069-3

**Published:** 2021-04-15

**Authors:** Yahui Ding, Huier Gao, Yu Zhang, Ye Li, Neil Vasdev, Yingdai Gao, Yue Chen, Quan Zhang

**Affiliations:** 1 State Key Laboratory of Medicinal Chemical Biology, College of Pharmacy and Tianjin Key Laboratory of Molecular Drug Research, Nankai University, Haihe Education Park, 38 Tongyan Road, Tianjin, 300353 People’s Republic of China; 2 State Key Laboratory of Experimental Hematology, Institute of Hematology and Hospital of Blood Diseases, Chinese Academy of Medical Sciences and Peking Union Medical College, Tianjin, 300020 People’s Republic of China; 3 Division of Nuclear Medicine and Molecular Imaging, Gordon Center for Medical Imaging, Massachusetts General Hospital, 55 Fruit St., Boston, MA 02114 USA; 4 Department of Radiology, Harvard Medical School, 55 Fruit St., Boston, MA 02114 USA

## Correction to: Journal of Hematology & Oncology (2016) 9:93 https://doi.org/10.1186/s13045-016-0327-5

The original article [1] contains an error in Fig. 4b, the panel of colonies of 5 µM Ara-C treatment was unintentionally duplicated onto the colonies of 2.5 µM alantolactone treatment. The error was mistakenly introduced in image integration when organizing the figures. However, the error has no bearing on the work’s scientific conclusions as the statistical results are based on the correct pictures. The authors apologize to the editor and readership of Journal of Hematology & Oncology for any inconvenience caused.

**Fig. 4:**
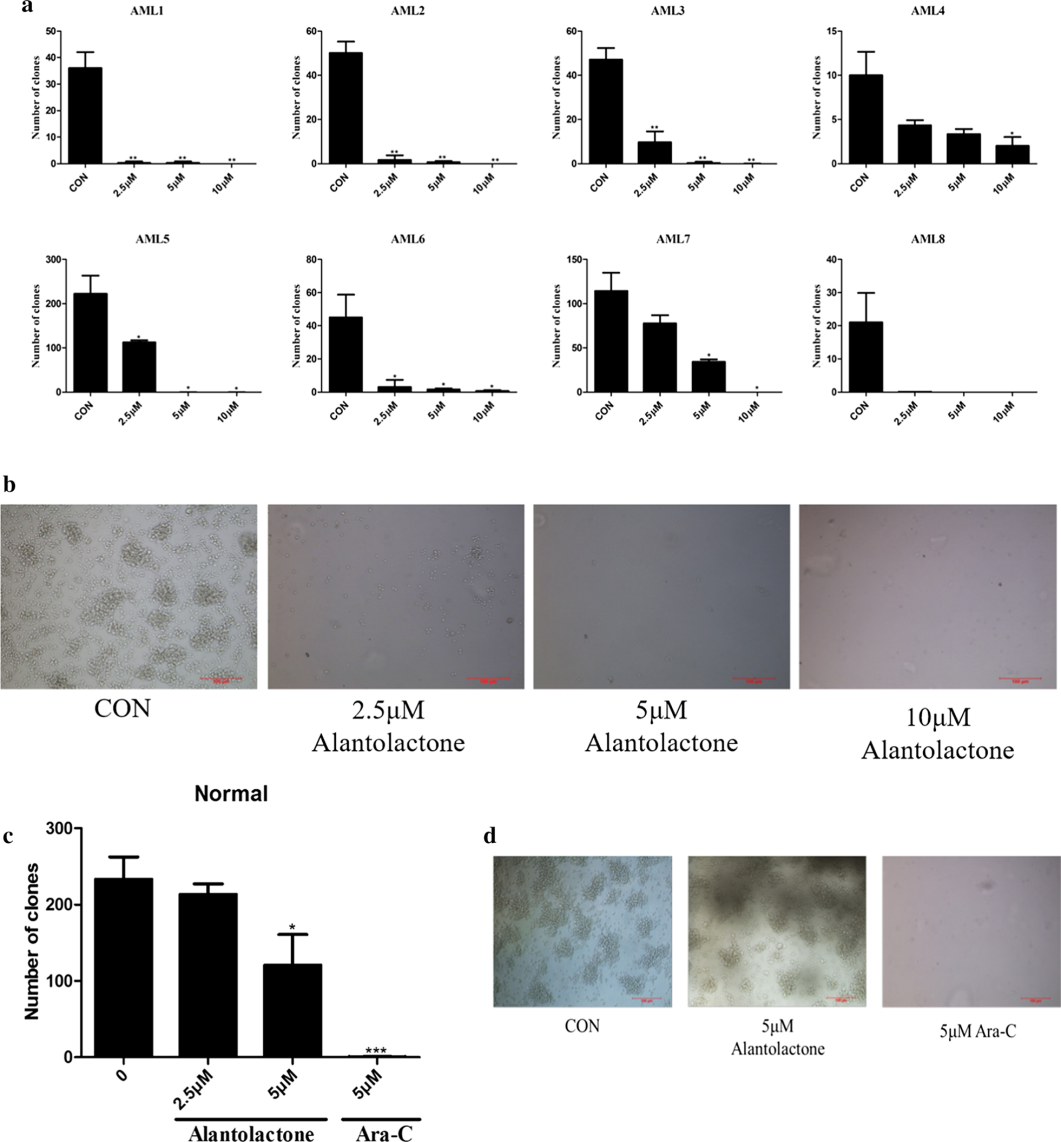
Alantolactone suppressed primary AML but not normal colony formation after 14 days in methocult H4434. **a** Alantolactone dramatically reduced the number of CFUs in primary AML cells from eight AML specimens with a dose-dependent manner. **b** The representative microscopy images of CFUs in primary AML cells were shown. **c** Alantolactone showed little effect on the CFUs in normal hematopoietic cells and Ara-C treatment led to notable reduction of colonies from normal donors. **d** The representative microscopy images of CFUs in normal hematopoietic cells were shown. *P < 0.05, **P < 0.01, ***P < 0.0001
