# Supplementary material for: Alantolactone selectively ablates acute myeloid leukemia stem and progenitor cells
Source: J Hematol Oncol. 2016 Sep 22;9:93. doi: 10.1186/s13045-016-0327-5 (PMC5034521; doi:10.1186/s13045-016-0327-5)
Supplement: Additional file 1: — Copies of NMR, MS, HPLC spectra of alantolactone and copies of NMR spectra of DMA-alantolactone. (DOCX 533 kb) [file 13045_2016_327_MOESM1_ESM.docx]

**Alantolactone Selectively Ablates Acute Myeloid Leukemia Stem and Progenitor Cells**

Yahui Ding^1^, Huier Gao^2^, Yu Zhang^2^, Ye Li^1^, Neil Vasdev^3^, Yingdai Gao^2,^*, Yue Chen^1,^*, Quan Zhang^1,^*

^1^*State Key Laboratory of Medicinal Chemical Biology, College of Pharmacy and Tianjin Key Laboratory of Molecular Drug Research, Nankai University, Haihe Education Park, 38 Tongyan Road, Tianjin 300353, People’s Republic of China*

^2^*State Key Laboratory of Experimental Hematology, Institute of Hematology and Hospital of Blood Diseases, Chinese Academy of Medical Sciences and Peking Union Medical College, Tianjin 300020, People’s Republic of China*

^3^*Division of Nuclear Medicine and Molecular Imaging, Gordon Center for Medical Imaging, Massachusetts General Hospital & Department of Radiology, Harvard Medical School, 55 Fruit St., Boston, MA, 02114, USA*

**Supplementary Information**

1. Copies of NMR, MS, HPLC spectra of alantolactone……………………………S3

2. Copies of NMR spectra of DMA-alantolactone…………………………………S5

**1. Copies of NMR, MS, HPLC spectra of alantolactone**

^1^H NMR (400 MHz, CDCl_3_) δ 6.18 (d, *J* = 1.6 Hz, 1H), 5.60 (s, 1H), 5.13 (d, *J* = 4.0 Hz, 1H), 4.82 (m, 1H), 3.56 (m, 1H), 2.43 (m, 1H), 2.09 (dd, *J* = 14.9, 2.4 Hz, 1H), 1.79 (m, 1H), 1.62 – 1.49 (m, 4H), 1.46 – 1.38 (m, 1H), 1.18 (s, 3H), 1.13 (dd, *J* = 13.4, 3.5 Hz, 1H), 1.08 (d, *J* = 7.6 Hz, 3H); ^13^C NMR (101 MHz, CDCl_3_) δ 170.42, 149.02, 139.80, 121.62, 118.74, 76.41, 42.62, 41.69, 39.46, 37.55, 32.68, 32.62, 28.54, 22.51, 16.71.


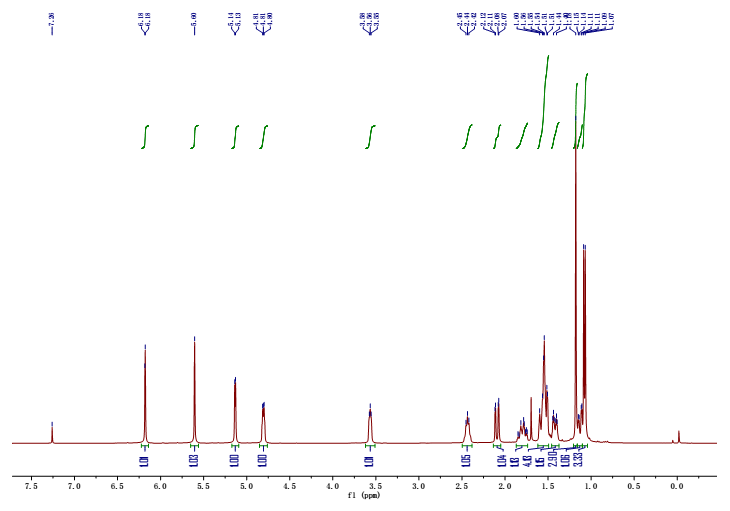


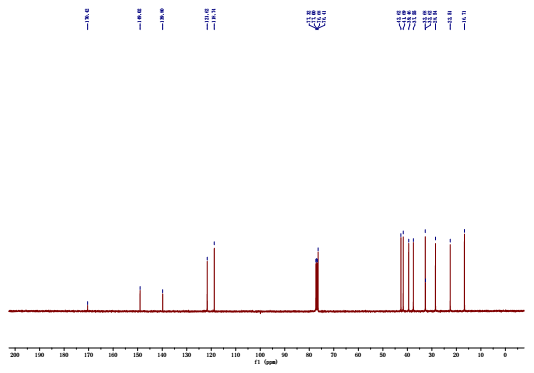

Agilent 1200 series HPLC system, Thermo hypersil BDS C18 colum (5 μm, 4.6х150 mm), 20%-100% MeOH in H_2_O over 8.0 min followed by 100% MeOH to 13.0 min, 1.0 mL/min, 25 ºC

**2. Copies of NMR spectra of DMA-alantolactone**


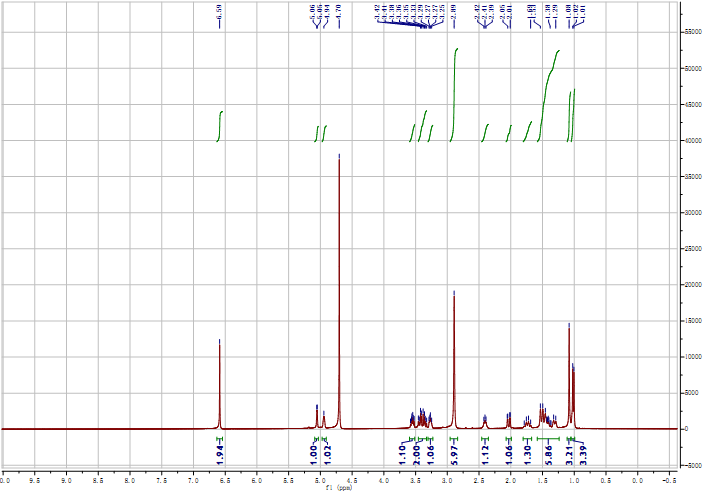


^1^H NMR of DMA-alantolactone

^13^C NMR of DMA-alantolactone
